# Extracellular vesicles from human urine-derived stem cells prevent osteoporosis by transferring CTHRC1 and OPG

**DOI:** 10.1038/s41413-019-0056-9

**Published:** 2019-06-26

**Authors:** Chun-Yuan Chen, Shan-Shan Rao, Yi-Juan Tan, Ming-Jie Luo, Xiong-Ke Hu, Hao Yin, Jie Huang, Yin Hu, Zhong-Wei Luo, Zheng-Zhao Liu, Zhen-Xing Wang, Jia Cao, Yi-Wei Liu, Hong-Ming Li, Yang Chen, Wei Du, Jiang-Hua Liu, Yan Zhang, Tuan-Hui Chen, Hao-Ming Liu, Ben Wu, Tao Yue, Yi-Yi Wang, Kun Xia, Peng-Fei Lei, Si-Yuan Tang, Hui Xie

**Affiliations:** 1 Department of Orthopedics, Xiangya Hospital, Central South University, Changsha, Hunan 410008 China; 2 Movement System Injury and Repair Research Center, Xiangya Hospital, Central South University, Changsha, Hunan 410008 China; 3 Xiangya School of Nursing, Central South University, Changsha, Hunan 410013 China; 4 Department of Sports Medicine, Xiangya Hospital, Central South University, Changsha, Hunan 410008 China; 5 Department of Rehabilitation, Xiangya Hospital, Central South University, Changsha, Hunan 410008 China; 6 Hunan Key Laboratory of Organ Injury, Aging and Regenerative Medicine, Changsha, Hunan 410008 China; 7 Hunan Key Laboratory of Bone Joint Degeneration and Injury, Changsha, Hunan 410008 China; 8 National Clinical Research Center for Geriatric Disorders, Xiangya Hospital, Central South University, Changsha, Hunan 410008 China

**Keywords:** Osteoporosis, Osteoporosis

## Abstract

Osteoporosis is a debilitating bone disease affecting millions of people. Here, we used human urine-derived stem cells (USCs), which were noninvasively harvested from unlimited and easily available urine, as a “factory” to obtain extracellular vesicles (USC-EVs) and demonstrated that the systemic injection of USC-EVs effectively alleviates bone loss and maintains bone strength in osteoporotic mice by enhancing osteoblastic bone formation and suppressing osteoclastic bone resorption. More importantly, the anti-osteoporotic properties of USC-EVs are not notably disrupted by the age, gender, or health condition (with or without osteoporosis) of the USC donor. Mechanistic studies determined that collagen triple-helix repeat containing 1 (CTHRC1) and osteoprotegerin (OPG) proteins are enriched in USC-EVs and required for USC-EV-induced pro-osteogenic and anti-osteoclastic effects. Our results suggest that autologous USC-EVs represent a promising novel therapeutic agent for osteoporosis by promoting osteogenesis and inhibiting osteoclastogenesis by transferring CTHRC1 and OPG.

## Introduction

Bone homeostasis is maintained by an orchestrated balance between bone destruction by osteoclasts and bone rebuilding by osteoblasts.^1,2^ Osteoporosis is a systemic skeletal disorder that occurs when bone breakdown exceeds new bone formation.^2^ Thus, therapeutic strategies designed to suppress osteoclastic bone resorption and promote osteoblastic bone formation will be beneficial for osteoporosis prevention and treatment.

Stem cell transplantation has promising therapeutic potential in treating various diseases. Growing evidence indicates that most transplanted stem cells do not engraft into the organs of recipient animals, and a single injection of stem cells is sufficient to alleviate disease phenotypes and maintain therapeutic efficacy for a long time,^3,4^ which suggests that the beneficial effects of these cells may be exerted by paracrine molecules. Stem cells are capable of releasing different types of extracellular vesicles (EVs), such as exosomes, microvesicles, etc. These EVs are classified on the basis of their subcellular origin, size, surface marker expression profile, and purification methods.^5–7^ Exosomes, 40–150 -nm EVs that originate from endosomal multivesicular bodies, are important paracrine mediators that transfer functional proteins and nucleic acids to target cells to evoke regenerative responses.^7–9^ These vesicles, with fewer safety concerns compared with stem cells after administration, provide researchers with a novel mechanism to stimulate bone formation.^10^ Since the term exosomes in most of the published articles is generally used to refer to small EVs without determining the intracellular origin,^7^ we thus chose here to use the generic term EVs, independent of the term used in the referenced articles. The local transplantation of EVs from mesenchymal stem cells (MSCs) from human-induced pluripotent stem cells (hiPSCs) is effective in repairing bone defects in ovariectomy-induced osteoporotic rats by promoting osteogenesis and angiogenesis.^11^ The intravenous injection of EVs from healthy mouse bone marrow-derived MSCs (BMSCs) has been found to rescue the impaired osteogenic potential of BMSCs and ameliorate the osteoporotic phenotype in mouse models of systemic lupus erythematosus (SLE) via the delivery of Fas protein.^3^ However, the application of iPSCs is often limited by genetic and epigenetic variations,^12^ and obtaining MSCs from the bone marrow is a painful and invasive procedure.^13^ Hence, identifying a simple, safe, and convenient new source of stem cells to collect EVs for bone remodeling and regeneration is needed.

One such alternative source is urine, which can be harvested by a safe, simple, noninvasive, low-cost, and easily repeatable procedure.^14^ We and others have confirmed that human urine contains abundant stem cells with MSC-like biological properties.^9,15^ We used human urine-derived stem cells (USCs) as a “factory” to generate EVs (USC-EVs) and found that USC-EVs could enhance skin cell function and accelerate the healing process of skin wounds in diabetic mice.^9^ Guan et al. reported the ability of USCs seeded onto a β-tricalcium phosphate scaffold to induce bone healing in rats with femoral defects.^16^ Considering the important role of EVs in cell activity,^3,17^ we hypothesized that the direct administration of USC-EVs may promote bone formation and prevent the development of osteoporosis. To address this hypothesis, we explored the impact of USC-EVs on bone formation and strength in mouse models of osteoporosis. In addition, the effects of USC-EVs on osteogenic activity and osteoclast formation were also evaluated. Furthermore, the molecules that may mediate USC-EV function in these processes were screened based on proteomic data, and their roles in the USC-EV-induced modulation of osteogenesis and osteoclastogenesis were assessed.

## Results

### USC-EVs are transported to the bone to enhance bone mass and strength in osteoporotic mice

**Fig. 1:**
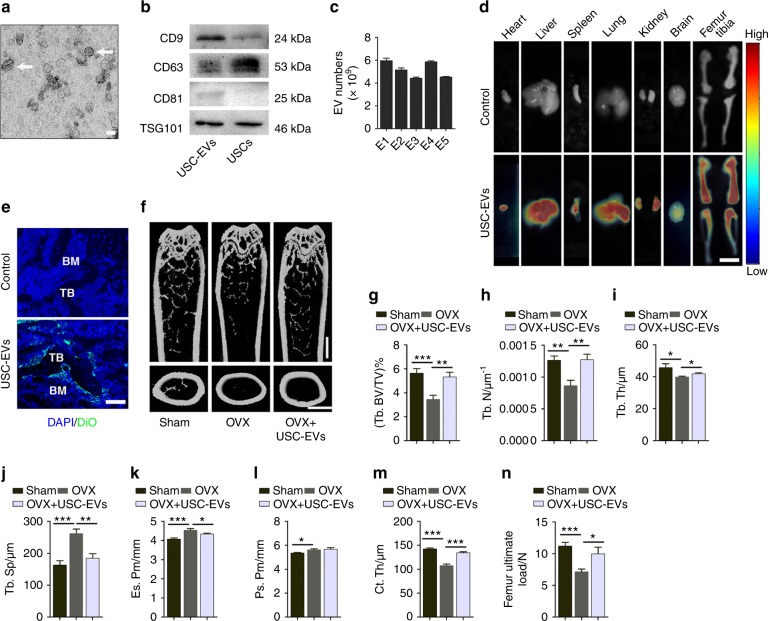
USC-EVs are transported to the bone to enhance bone mass and strength in OVX-induced osteoporotic mice. **a** Morphology of USC-EVs under transmission electron microscopy. Scale bar: 50 nm. **b** Western blot analysis of exosomal markers in USC-EVs and USCs. **c** EV numbers of 100 μg USC-EVs from five different batches (E1, E2, E3, E4, and E5) were assessed by an EXOCET Exosome Quantitation kit. **d** Ex vivo fluorescent imaging of heart, liver, spleen, lungs, kidneys, brain, femurs, and tibias from vehicle-treated control OVX mice and OVX mice intravenously injected with the DiR-labeled USC-EVs for 3 h. Scale bar: 6 mm. **e** Fluorescence microscopy analysis of femur tissue sections from vehicle-treated OVX mice and OVX mice intravenously injected with the DiO-labeled USC-EVs for 3 h. TB: trabecular bone; BM: bone marrow. Scale bar: 50 µm. **f** Representative μCT images of femora. Scale bars: 1 mm. **g**–**m** Quantitative μCT analysis of the trabecular bone volume fraction (Tb. BV/TV; **g**), trabecular number (Tb. N; **h**), trabecular thickness (Tb. Th; **i**), trabecular separation (Tb. Sp; **j**), endosteal perimeter (Es. Pm; **k**), periosteal perimeter (Ps. Pm; **l**), and cortical thickness (Ct. Th; **m**). *n* = 10 per group. **n** Three-point bending measurement of femur ultimate load. *n* = 5 per group. The data are shown as the mean ± SD. *P < 0.05, **P < 0.01, ***P < 0.001

Supplementary Fig. 1 shows USCs obtained from a healthy adult woman (28-years old). These cells exhibited typical characteristics of MSCs, including a spindle-like morphology (Supplementary Fig. 1a), the capacity to differentiate toward osteoblasts, adipocytes, and chondrocytes (Supplementary Fig. 1b), and MSC surface marker expression profiles (CD90^+^, CD73^+^, CD45^−^, CD44^+^, CD34^−^, and CD29^+^; Supplementary Fig. 1c). USC-EVs showed a cup- or sphere-like morphology with diameters of ~50 nm under a transmission electron microscope (Fig. 1a) and expressed TSG101, CD81, CD63, and CD9 (Fig. 1b), which demonstrated their exosome identity. Particle number test using an EXOCET Exosome Quantitation Kit revealed that the vesicle numbers were not exactly the same in 100 μg samples of USC-EVs from different batches but were mostly in the range of (4–6) × 10^9^ vesicles (Fig. 1c).

Ovariectomized (OVX) animals have been widely used as experimental models of postmenopausal osteoporosis.^18^ To investigate the potential of USC-EVs as effective agents for postmenopausal osteoporosis treatment, we first generated OVX mice and explored whether USC-EVs could be transported into bone tissues after intravenous administration. Previous studies have demonstrated that for each mouse, the injection of 100 μg of EVs only one time^3,19,20^ or weekly for 2 months^21^ is sufficient to induce significant pro-regenerative effects in recipient mice. Based on these studies, a dose of 100 μg of USC-EVs per mouse was chosen for our in vivo experiments. We labeled the USC-EVs obtained from 28-year-old healthy adult woman-derived USCs with DiR iodide and used a fluorescence tomography imaging system to detect the distribution of DiR-labeled USC-EVs in the heart, liver, spleen, lungs, kidneys, brain, femurs, and tibias of OVX mice after intravenous injection for 3 h. The results showed high fluorescent signals in the highly vascularized organs, including the heart, liver, spleen, lungs, and kidneys (Fig. 1d). Very high fluorescent signals could also be detected in the mouse femurs and tibias, indicating that large numbers of USC-EVs are transported into bone tissues (Fig. 1d). The fluorescent signals were much lower in the mouse brain compared with those in other organs (Fig. 1d), suggesting that relatively low levels of USC-EVs enter into the brain. We also labeled USC-EVs with DiO and assessed the green fluorescent signals in the above-described tissues after the injection of DiO-labeled USC-EVs for 3 h by using a fluorescence microscope. The liver tissue sections were costained with an endothelial marker CD31 to detect whether USC-EVs can enter into endothelial cells. The results confirmed USC-EV uptake by the endothelium and cells in the mouse heart, liver, spleen, lungs, kidneys, brain, femurs, and tibias (Fig. 1e; Supplementary Fig. 2). In bone tissues, USC-EVs were predominantly accumulated on trabecular bone surfaces, where active bone formation or bone remodeling often occurs (Fig. 1e; Supplementary Fig. 2). These findings suggest that USC-EVs may accumulate in the bone to modulate bone metabolism.

To demonstrate the effects of USC-EVs in a mouse model of postmenopausal osteoporosis, OVX mice were intravenously injected with USC-EVs obtained from 28-year-old healthy adult woman-derived USCs or with the vehicle (PBS). As an observation period of 1 or 2 months is usually selected to evaluate the effects of a new treatment for postmenopausal osteoporosis in animals,^1,18^ we conducted EV treatments weekly for 2 months to assess the effects of USC-EVs on animal models of postmenopausal osteoporosis in our study. The mice that underwent OVX had much smaller uterus sizes (Supplementary Fig. 3a) and lower uterus weights (Supplementary Fig. 3b) relative to those of Sham mice, which determined the success of the OVX surgery. Microcomputed tomography (μCT) analysis indicated that the femurs of OVX mice showed notable osteoporotic phenotypes, as indicated by significantly reduced trabecular bone volume fraction (Tb. BV/TV), trabecular number (Tb. N), trabecular thickness (Tb. Th), and cortical thickness (Ct. Th), as well as increased endosteal perimeter (Es. Pm) and trabecular separation (Tb. Sp), compared with those of Sham mice (Fig. 1f–m). Particularly, the increase in Es. Pm suggests the enhancement of bone resorption in OVX mice. Surprisingly, at 8 weeks after EV injection, all altered parameters induced by OVX were rescued (Fig. 1f–m), implying that USC-EVs are capable of suppressing the excessive osteoclastic activity or/and augmenting the osteoblastic bone formation to maintain bone mass. A three-point bending test showed that OVX induced a marked decrease in the femur ultimate load value, which represents bone strength; however, the reduction in bone strength was prevented by USC-EVs (Fig. 1n).

We also verified the therapeutic effects of USC-EVs from the above-described donor-derived USCs in mouse models of senile osteoporosis and tail suspension-induced hind-limb disuse osteoporosis. As an observation period of 3 months has been previously chosen to test the effects of a new intervention for senile osteoporosis in animals, we performed EV treatment weekly for 3 months to evaluate the impact of USC-EVs on mouse models of senile osteoporosis. Since previous evidence has revealed that significant bone loss occurs in the hind limb of animals after 2- or 3-week tail suspension,^22,23^ we conducted EV treatment twice a week for 3 weeks in mice subjected to tail suspension to evaluate the effects of USC-EVs on disuse osteoporosis. The μCT analysis and three-point bending test, respectively, revealed that the systemic injection of USC-EVs significantly enhanced trabecular and cortical bone mass (Supplementary Fig. 4a–d) and bone strength (Supplementary Fig. 4e, f) in aging and hind-limb unloading-induced osteoporotic mice. Increases in the periosteal perimeter (Ps. Pm) and Ct. Th induced by USC-EVs in aging mice suggest that USC-EVs have the ability to enhance osteoblastic bone formation. Altogether, these data suggest that USC-EVs are able to inhibit the bone loss induced by various factors and maintain bone strength.

### USC-EVs enhance osteogenic activities and inhibit osteoclast formation

**Fig. 2:**
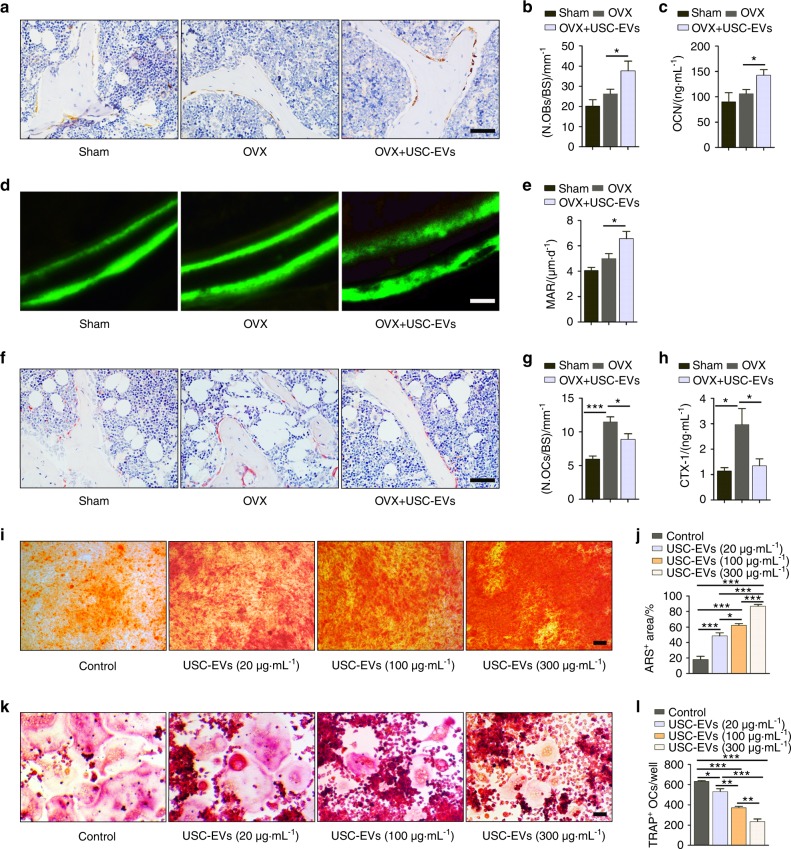
USC-EVs promote osteogenesis and inhibit osteoclast formation. **a**, **b** Representative OCN immunohistochemical staining images (**a**) with quantification of the number of osteoblasts (N. OBs; **b**) on trabecular bone surface (BS) of femoral metaphysis. Scale bar: 50 μm. *n* = 3 per group. **c** ELISA of the serum concentration of OCN. *n* = 5 per group. **d**, **e** Representative images of calcein double labeling of trabecular bone (**d**) with quantification of mineral apposition rate (MAR; **e**). Scale bar: 25 μm. *n* *=* 3 per group. **f**, **g** Representative TRAP staining images (**f**) and quantitative analysis of the number of osteoclasts (N. OCs; **g**). Scale bar: 50 μm. *n* = 3 per group. **h** ELISA of the serum concentration of CTX-I. *n* = 5 per group. **i**, **j** Alizarin red S (ARS) staining of mineralized nodules of MSCs receiving different treatments under osteogenic inductive conditions (**i**). The percentages of ARS positively stained areas were measured (**j**). Scale bar: 100 μm. *n* = 3 per group. **k**, **l** Osteoclast differentiation of RAW264.7 cells visualized by TRAP staining (**k**). The numbers of TRAP^+^ multinucleated (> 3 nuclei) osteoclasts in each well of a 48-well plate were counted (**l**). Scale bar: 50 μm. *n* = 3 per group. The data are shown as the mean ± SD. *P < 0.05, **P < 0.01, ***P < 0.001

Next, we determined the effects of USC-EVs on osteogenesis and osteoclastogenesis in OVX mice. Immunohistochemical staining for osteocalcin (OCN; a marker of osteogenesis^2^) showed slightly enhanced numbers of OCN-positive osteoblasts on the surface of trabecular bones in OVX mice relative to Sham mice, whereas the injection of USC-EVs significantly increased the number of osteoblasts in OVX mice (Fig. 2a, b). The administration of USC-EVs to OVX mice also induced a prominent increase in OCN secretion, as determined by the serum levels of OCN using enzyme-linked immunosorbent assay (ELISA) (Fig. 2c). Calcein double labeling confirmed that compared with vehicle-treated OVX mice, OVX mice treated with USC-EVs had a prominent enhanced capacity to generate new mineralized bone, as indicated by mineral apposition rate (MAR) values (Fig. 2d, e). Tartrate-resistant acid phosphatase (TRAP) staining revealed a much larger quantity of osteoclasts on the surface of trabecular bones in mice that underwent OVX, but osteoclast numbers were markedly decreased in OVX mice treated with USC-EVs (Fig. 2f, g). Consistently, ELISA showed that the serum level of the bone resorption marker C-terminal telopeptides of type I collagen (CTX-I) was remarkably enhanced in OVX mice compared with Sham mice, but its upregulation was blocked by USC-EVs (Fig. 2h). USC-EVs also enhanced OCN secretion and reduced CTX-I production in mouse models of senile osteoporosis (Supplementary Fig. 4g) and hind limb disuse osteoporosis (Supplementary Fig. 4h), which further confirmed the pro-osteogenic and anti-osteoclastic abilities of these molecules.

We then evaluated the direct impact of USC-EVs on osteogenic differentiation and osteoclast formation in vitro. We labeled USCs with a red lipophilic dye (DiL) to assess the cellular uptake of USC-EVs. Fluorescence microscopy showed that USC-EVs could be transferred to the perinuclear region of mouse MSCs and osteoclast progenitor RAW264.7 cells after incubation for 3 h (Supplementary Fig. 5a). For in vitro experiments, 1–200 μg·mL^−1^ EVs have been used in previous published studies.^3,20,21^ We compared the effects of 20, 100, and 300 μg·mL^−1^ USC-EVs on the osteogenesis of mouse bone marrow stromal cells (MSCs) and the osteoclastic differentiation of osteoclast progenitor RAW264.7 cells. Alizarin Red S (ARS) and TRAP staining revealed that USC-EVs markedly enhanced the calcium nodule formation of MSCs and reduced the osteoclast formation of RAW264.7 cells in a dose-dependent manner (Fig. 2i–l). We selected a high dose of USC-EVs (300 μg·mL^−1^) for subsequent in vitro assays. Quantitative real-time PCR (qRT-PCR) analysis showed that USC-EV treatment increased the mRNA levels of genes related to osteogenesis, including *Ocn*, *Alp*, and *Runx2*, in differentiated MSCs (Supplementary Fig. 5b). Direct treatment with USC-EVs also inhibited the expression of osteoclast formation-related genes (*Trap*, *Mmp9*, *Ctsk*, *Oscar*, *Ocstamp*, and *Atp6v0d2*) (Supplementary Fig. 5c). These findings determine the pro-osteogenic and anti-osteoclastic properties of USC-EVs.

### Multiple donor-derived USC-EVs are able to exert anti-osteoporotic effects

**Fig. 3:**
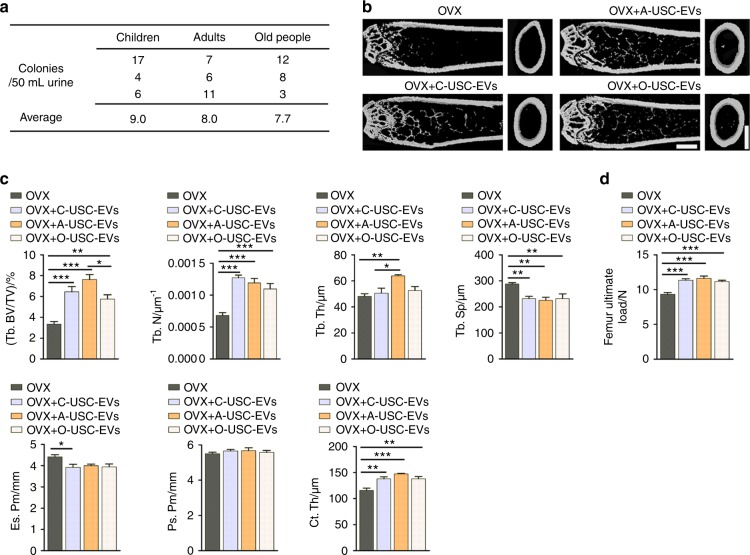
Multiple donor-derived USC-EVs are able to exert anti-osteoporotic effects on OVX mice. **a** The numbers of USC colonies obtained from three healthy children, three healthy adults and three old people (one healthy man and two postmenopausal osteoporotic women). **b**, **c** Representative μCT images (**b**) and quantitative μCT analysis of trabecular and cortical bone microarchitecture (**c**) in femora from Sham, OVX, OVX + Y-USC-EVs, OVX + A-USC-EVs, and OVX + O-USC-EVs mice. C: children; A: adults; O: old people. Scale bars: 1 mm. *n* = 7–10 per group. **d** Three-point bending measurement of femur ultimate load. *n* = 7–10 per group. The data are shown as the mean ± SD. *P < 0.05, **P < 0.01, ***P < 0.001

**Fig. 4:**
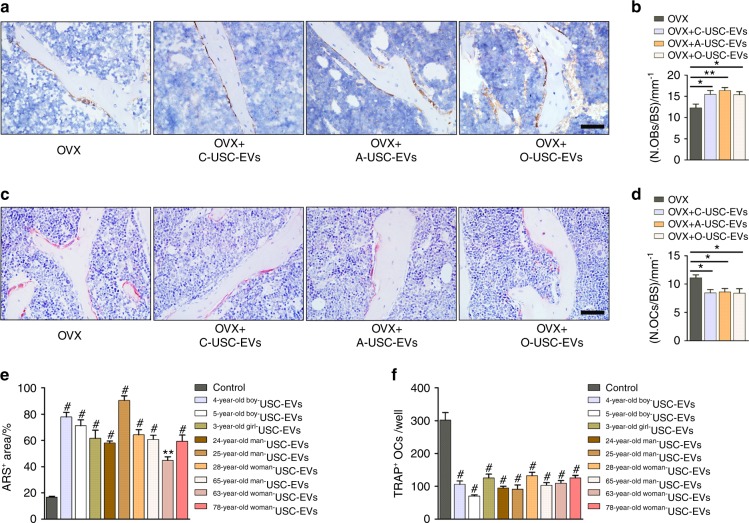
Multiple donor-derived USC-EVs are able to promote osteogenesis and inhibit osteoclast formation. **a**, **b** Representative images of OCN immunostaining (**a**) with quantification of the number of OBs (**b**). Scale bar: 50 μm. *n* = 3 per group. **c**, **d** Representative TRAP staining images (**c**) and quantitative analysis of the number of OCs (**d**). Scale bar: 50 μm. *n* = 3 per group. **e** The percentages of ARS positively stained areas in MSCs receiving different treatments under osteogenic inductive conditions. *n* = 3 per group. **f** Quantification of osteoclast formation in RAW264.7 cells receiving different treatments under osteoclastic induction. *n* = 3 per group. The data are shown as the mean ± SD. *P < 0.05, **P < 0.01, ***P < 0.001

Studies revealed that USCs can be obtained from human urine, regardless of age or health state (except in patients suffering from anuria or urinary tract infection).^9,14,15^ We harvested USCs (8.2 ± 4.4 colonies per 50 mL urine; Fig. 3a) from three healthy children (two boys and one girl; 3−5-years old), three healthy adults (two men and one woman; 24−28-years old) and three elderly individuals (a 65-year-old healthy man and two postmenopausal osteoporotic woman aged 63 and 78 years, respectively). USCs from a 5-year-old healthy boy, 24-year-old healthy adult man, and 63-year-old osteoporotic postmenopausal woman were selected for assessing whether their derived EVs could reverse the osteoporotic phenotypes in OVX mice. Significant reductions in uterus sizes (Supplementary Fig. 6a) and weights (Supplementary Fig. 6b) were observed in all OVX mice relative to Sham mice. Notably, as indicated by µCT and three-point bending test, USC-EVs from these donors could effectively enhance bone mass (Fig. 3b, c) and bone strength (Fig. 3d) in OVX mice. OCN immunostaining and TRAP staining, respectively, revealed that these USC-EVs could promote the production of osteoblasts (Fig. 4a, b) and repress the formation of osteoclasts (Fig. 4c, d) after administration to OVX mice. The abilities of the USC-EVs from the nine donors described above with different ages, genders, and health conditions to promote the osteogenesis of MSCs and inhibit the osteoclastogenesis of RAW264.7 cells were verified by ARS (Fig. 4e) and TRAP staining (Fig. 4f), respectively. These in vivo and in vitro findings suggest promising prospects for the future utilization of autologous USC-EVs for osteoporosis therapy.

### Enrichment of pro-osteogenic and anti-osteoclastic proteins in USC-EVs

**Fig. 5:**
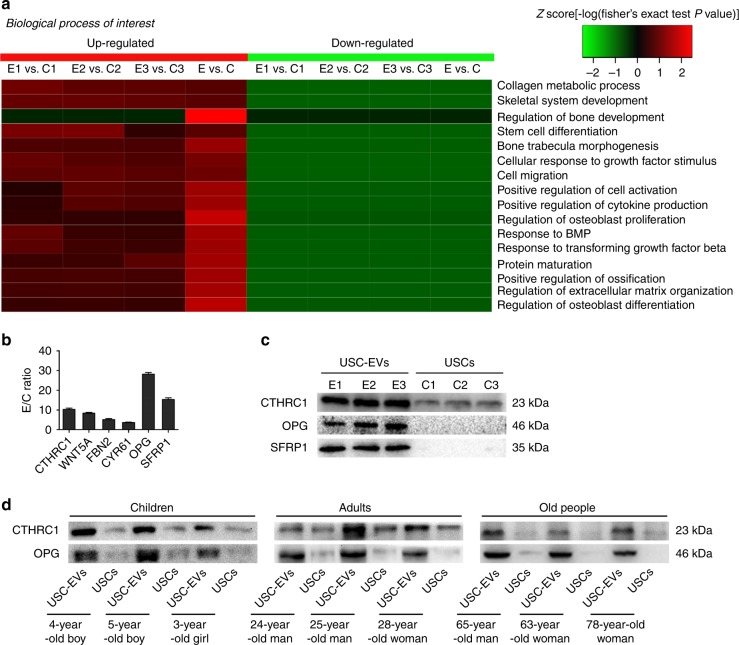
Enrichment of pro-osteogenic and anti-osteoclastic proteins in USC-EVs. **a** When compared with USCs, USC-EVs were highly enriched in the proteins that are involved in the regulation of multiple biological processes related to bone formation and growth. C: USCs; E: USC-EVs. *n* = 3 per group. **b** The ratio of expression of a class of pro-osteogenic or/and anti-osteoclastic proteins in USC-EVs compared with that in USCs. *n* = 3 per group. **c** The upregulation of CTHRC1, OPG, and SFRP1 in USC-EVs relative to USCs was verified by western blotting. **d** Western blot analysis of the protein levels of CTHRC1 and OPG in USC-EVs and USCs from different donors

In our previous study, we compared the protein expression profiles in 28-year-old healthy adult woman-derived USCs and their USC-EVs using ITRAQ-based proteomic analysis.^9^ All identified proteins in USC-EVs and USCs were shown in this published study.^9^ Herein, Fig. 5a shows that USC-EVs contained abundant proteins, which have roles in regulating bone formation and bone growth-related biological processes, such as stem cell differentiation, osteoblast proliferation and differentiation, bone development, bone trabecula morphogenesis, ossification, and extracellular matrix organization. Figure 5b reveals the expression ratio of a cluster of pro-osteogenic or/and anti-osteoclastic proteins in USC-EVs relative to USCs. Among these proteins, collagen triple-helix repeat containing 1 (CTHRC1), Wnt family member 5 A (WNT5A), fibrillin-2 (FBN2), and cysteine-rich angiogenic inducer 61 (CYR61) are capable of promoting osteogenesis.^24–27^ CTHRC1, with the highest E/C ratio (10.27 ± 1.21-fold) among the USC-EV-enriched pro-osteogenic proteins (Fig. 5b), has been shown to be able to stimulate osteoblast differentiation^24,27^ and inhibit osteoclast formation and function,^28^ suggesting a potential role for CTHRC1 as a mediator in the USC-EV-induced promotion of osteogenesis and inhibition of osteoclastogenesis. Tumor necrosis factor receptor superfamily member 11b [TNFRSF11B; also called osteoprotegerin (OPG)] and secreted frizzled SF-related protein-1 (SFRP1) are also able to suppress osteoclastic differentiation.^29,30^ OPG, which can act as a decoy receptor for RANKL to interrupt RANKL-induced osteoclast formation,^29^ showed the highest E/C ratio (28.11 ± 0.36-fold) among the USC-EV-enriched anti-osteoclastic proteins (Fig. 5b). Thus, we selected CTHRC1 and OPG as candidate proteins to explore the mechanism through which USC-EVs exert pro-osteogenic and anti-osteoclastic functions. As evidenced by western blotting, although CTHRC1 and OPG were differently expressed in the USC-EVs from the above-described nine donors with different ages, genders, and health conditions, these two proteins, especially OPG, were highly enriched in USC-EVs compared with their parent USCs (Fig. 5c, d). The upregulation of SFRP1 in USC-EVs relative to USCs was also verified by western blotting (Fig. 5c), which showed results consistent with the proteomic data.

### CTHRC1 and OPG contribute to USC-EV-induced increases in bone mass and strength

**Fig. 6:**
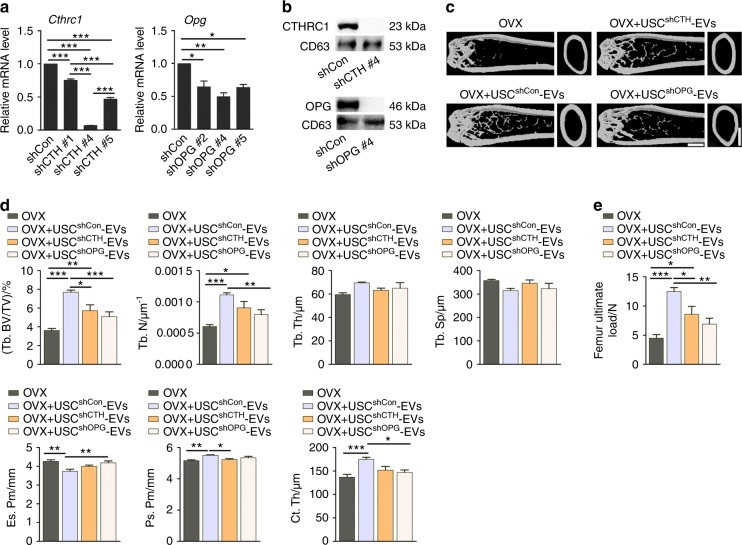
CTHRC1 and OPG contribute to USC-EV-induced increases in bone mass and strength. **a** The inhibitory efficiency of shRNAs targeting CTHRC1 and OPG was verified by qRT-PCR analysis. shCTH: shCTHRC1. **b** The deficiency of CTHRC1 and OPG in USC-EVs was verified by western blotting. **c**, **d** Representative μCT images (**c**) and quantitative μCT analysis of trabecular and cortical bone microarchitecture (**d**) in femora. USC^shCTH^-EVs: USC^shCTHRC1 #4^-EVs; USC^shOPG^-EVs: USC^shOPG #4^-EVs. Scale bars: 1 mm. *n* = 6–8 per group. **e** Three-point bending measurement of the femur ultimate load. *n* = 6–8 per group. The data are shown as the mean ± SD. *P < 0.05, **P < 0.01, ***P < 0.001

To determine the role of CTHRC1 and OPG in USC-EV-induced bone protective effects, we downregulated the expression of CTHRC1 and OPG in USCs from the above-mentioned 28-year-old woman using three different shRNAs per target gene. qRT-PCR confirmed the highest efficiency of shCTHRC1 #4 and shOPG #4 in inhibition of CTHRC1 and OPG, respectively (Fig. 6a). USCs transfected with these shRNAs or with the nontarget control shRNA (shCon) were then used to produce EVs for the following experiments. Western blot analysis confirmed the downregulation of CTHRC1 and OPG in USC^shCTHRC1 #4^-EVs and USC^shOPG #4^-EVs relative to USC^shCon^-EVs, respectively (Fig. 6b).

OVX mice were injected intravenously with USC^shCTHRC1 #4^-EVs, USC^shOPG #4^-EVs, USC^shCon^-EVs or vehicle (PBS) for 2 months. The reductions in uterus sizes and uterus weights were observed in OVX mice relative to Sham mice (Supplementary Fig. 6c, d). As expected, μCT analysis showed that knockdown of either of these two proteins in USCs, especially OPG, markedly suppressed their EV-mediated anti-osteoporotic effects, including the improved Tb. BV/TV, Tb. N, Ps. Pm or/and Ct. Th, as well as the decreased Es. Pm of the femur in OVX mice (Fig. 6c, d). The significantly lower ability of USC^shCTHRC1 #4^-EVs to enhance Ps. Pm than that of USC^shCon^-EVs suggests that the beneficial effect of USC-EVs on osteoblastic bone formation is reduced once CTHRC1 protein contents are decreased in USC-EVs. USC^shOPG #4^-EVs showed a comparable ability to enhance Ps. Pm but a much weaker ability to diminish Es. Pm relative to USC^shCon^-EVs, implying the downregulation of the ability of USC-EVs to suppress bone resorption when OPG is decreased in USC-EVs. Three-point bending test revealed that knockdown of CTHRC1 or OPG suppressed the capacity of USC-EVs to enhance bone strength in OVX mice, as assessed by femur ultimate load values (Fig. 6e). These data suggest that CTHRC1 and OPG are essential for the USC-EV-mediated prevention of the loss of bone mass and strength.

We photographed spleen samples from all control mice and the above-described osteoporosis mouse models receiving different treatments. The results showed that the mice treated with USC-EVs from different donors, USC^shCon^-EVs, USC^shCTHRC1 #4^-EVs, or USC^shOPG #4^-EVs, showed comparable spleen sizes compared with those of vehicle-treated control mice (Supplementary Fig. 7a–e). Hematoxylin and eosin (H&E) staining showed no marked histopathological changes, such as lymph node hyperplasia and inflammatory cell infiltration, in OVX mice subjected to EV injections (Supplementary Fig. 7f). In addition, we performed ELISA to detect the concentrations of pro-inflammatory factors (TNF-α, IL-6, IL-1α, and IL-1β) in serum from the above-described mouse models of osteoporosis. The results showed that USC-EV treatment only caused a statistically significant increase in the concentrations of serum IL-1β in OVX mice and TS-treated mice compared with the control mice, whereas the concentrations of serum IL-1α and IL-6 were reduced in these mice. Moreover, the aged mice treated with USC-EVs exhibited decreasing serum levels of IL-1α and IL-1β (Supplementary Fig. 7g–i). Collectively, these findings suggest that no obvious immune and inflammatory responses are induced in mice after the injection of USC-EVs.

### CTHRC1 and OPG contribute to the pro-osteogenic and anti-osteoclastic effects of USC-EVs

**Fig. 7:**
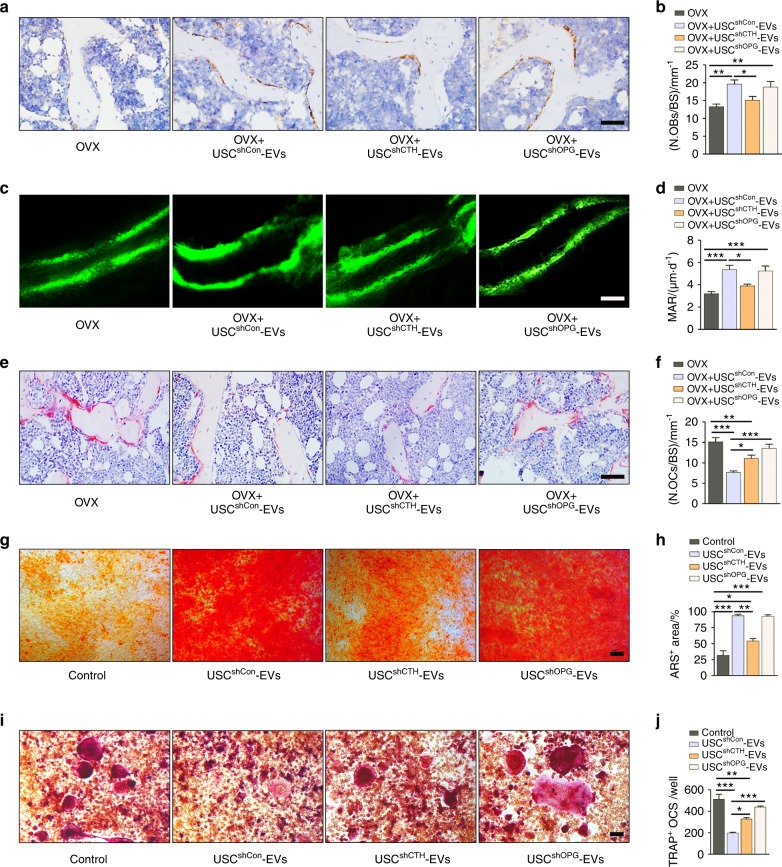
CTHRC1 and OPG contribute to the pro-osteogenic and anti-osteoclastic effects of USC-EVs. **a**, **b** Representative images of OCN immunostaining (**a**) with quantification of the number of OBs (**b**). Scale bar: 50 μm. *n* = 3 per group. **c**, **d** Representative images of calcein double labeling of trabecular bone (**c**) with quantification of MAR (**d**). Scale bar: 25 μm. *n* = 3 per group. **e**, **f** Representative TRAP staining images (**e**) and quantitative analysis of the number of OCs (**f**). Scale bar: 50 μm. *n* = 3 per group. **g**, **h** ARS staining of mineralized nodules of MSCs receiving different treatments (**g**). Scale bar: 100 μm. The percentages of ARS positively stained areas were measured (**h**). *n* = 3 per group. **i**, **j** Osteoclast differentiation of RAW264.7 cells visualized by TRAP staining (**i**). Scale bar: 50 μm. The numbers of TRAP^+^ osteoclasts per well were counted (**j**). *n* = 3 per group. The data are shown as the mean ± SD. *P < 0.05, **P < 0.01, ***P < 0.001

Finally, we determined whether USC-EVs exert anti-osteoporotic effects through CTHRC1- or OPG-mediated promotion of osteogenesis or/and inhibition of osteoclast formation. OCN immunohistochemical staining and calcein double labeling revealed that USC^shCTHRC1 #4^-EVs had significantly reduced capacity to enhance osteogenic responses (Fig. 7a, b) and bone mineralization (Fig. 7c, d) in OVX mice relative to that of USC^shCon^-EVs, suggesting a mediatory role of CTHRC1 in USC-EV-induced pro-osteogenic effects. OVX mice treated with USC^shOPG #4^-EVs exhibited comparable OCN expression in bone tissue (Fig. 7a, b) and comparable rates of bone mineralization (Fig. 7c, d) compared with those of mice treated with USC^shCon^-EVs, suggesting that the inhibition of OPG in USC-EVs does not disturb the pro-osteogenic effects of USC-EVs. TRAP staining of femur sections revealed that USCs^shCTHRC1 #4^-EVs and USCs^shOPG #4^-EVs exhibited lower abilities to inhibit the formation of osteoclasts in OVX mice when compared with those of USCs^shCon^-EVs (Fig. 7e, f), suggesting that both CTHRC1 and OPG contribute to the USC-EV-induced suppression of osteoclastogenesis.

Consistent with the in vivo data, the positive effects of USC-EVs on the osteogenic differentiation of cultured MSCs were remarkably inhibited when CTHRC1, but not OPG, was silenced in USCs, as indicated by lower levels of mineralized nodule formation in MSCs treated with USC^shCTHRC1 #4^-EVs compared with those treated with USC^shCon^-EVs (Fig. 7g, h). TRAP staining revealed that the inhibition of the expression of either CTHRC1 or OPG in USCs downregulated the ability of their EVs to prevent the osteoclastic differentiation of RAW264.7 cells (Fig. 7i, j). These data indicate that CTHRC1 partially mediates USC-EV-induced pro-osteogenic and anti-osteoclastic effects, whereas OPG only contributes to the anti-osteoclastic function of USC-EVs.

As OPG is a secreted protein that requires binding to soluble RANKL or RANKL on the cell surface to inhibit osteoclast differentiation,^29,31^ we further explored the location of this protein when USC-EVs were internalized by recipient cells. USCs were transfected with plasmids expressing OPG-enhanced green fluorescent protein (OPG-EGFP), and then these cells were used to produce USC-EVs containing OPG-EGFP (USC-EVs^OPG-EGFP^). Subsequently, we tested the uptake of USC-EVs^OPG-EGFP^ by osteoclast progenitor RAW264.7 cells. The results revealed the presence of OPG-EGFP (green fluorescence signals) mainly in the perinuclear region, cytoplasm, and cell membrane of RAW264.7 cells after treatment with USC-EVs^OPG-EGFP^ for 3 h (Supplementary Fig. 8). A few green signals were also observed in the extracellular region of RAW264.7 cells, suggesting the transfer of OPG-EGFP to the extracellular matrix of adherent recipient cells (Supplementary Fig. 8). The results may explain why OPG protein from USC-EVs can mediate the inhibitory effects of USC-EVs on the soluble RANKL-induced osteoclast differentiation of RAW264.7 cells and on osteoclast formation and bone loss in vivo.

## Discussion

Osteoporosis causes high rates of fractures and mortality and is a serious threat to the quality of life of aging individuals, particularly postmenopausal women.^32^ The current therapeutic options for osteoporosis are mainly agents that either suppress bone resorption or stimulate bone formation, such as bisphosphonates, denosumab, raloxifene, and teriparatide.^33^ However, the serious side effects of these drugs have led to an urgent need for exploring an alternative agent for osteoporosis treatment.^34–39^ MSC-based therapy has attracted considerable attention for treating osteoporosis^33,34^, and the direct use of their secreted EVs may be a safer and promising choice for therapeutic purposes.^40,41^ In this study, we used USCs that exhibited MSC-like properties and could be noninvasively harvested from unlimited and easily available human urine as the parent cell source to obtain EVs (USC-EVs) and determined that the systemic injection of USC-EVs effectively inhibited bone loss and increased bone strength in osteoporotic mice due to their dual effects on bone metabolism, which not only enhanced osteoblastic bone formation but also reduced osteoclastic bone resorption. Studies have revealed that BMSCs from osteoporotic bone have defects in intrinsic signals that impair the osteogenic differentiation capacity of BMSCs.^3,42,43^ The use of autologous BMSCs or adipose tissue-derived MSCs (AdMSCs) for osteoporosis therapy is also limited by the age-induced reduction of the total numbers of MSCs.^44,45^ In our study, we found that the yield of USC colonies was not significantly reduced in aged donors relative to children and adults. More importantly, the anti-osteoporotic properties of USC-EVs were not notably disturbed by the age, gender, or health condition (with or without osteoporosis) of the USC donor, as USC-EVs from a healthy boy, healthy adult man and woman, and postmenopausal osteoporotic woman could induce notable anti-osteoporotic effects. In vitro, the USC-EVs from nine donors of different ages, genders, and health conditions had the ability to promote osteogenesis and inhibit osteoclastogenesis. Our findings suggest a promising future prospect for autologous USC-EVs to be used as a new treatment for osteoporotic patients. That is, to obtain therapeutic EVs for osteoporosis treatment, osteoporotic patients may just need to collect a certain volume of their own urine to harvest USCs.

Nevertheless, if we can test the immunogenicity of USC-EVs, then it will be useful to evaluate their safety when the use of allogeneic or heterogeneic USC-EVs is required. In our study, we found that human USC-EVs did not evoke obvious immune responses in recipient mice, as revealed by comparable spleen sizes and histological structures between USC-EV-treated mice and vehicle-treated control mice. However, we did not test the levels of antibodies against USC-EVs in these mice and examined the immune responses of mice after the long-term use of these heterogeneic USC-EVs. Future studies are required to comprehensively and systematically assess the immunogenicity of USC-EVs.

EVs can transfer RNA and proteins to recipient cells and thereby alter their bioactivity.^7–9,17^ CTHRC1, a secreted protein highly conserved among vertebrates, was first identified in the injured arterial wall.^46^ Recent studies have reported the roles of CTHRC1 in bone metabolism. Kimura et al. showed that CTHRC1 is required for the osteogenic differentiation of cultured MSCs, and *Cthrc1* null mice have lower bone mass relative to the control mice due to reduced osteoblastic bone formation.^47^ Takeshita et al. revealed that CTHRC1 is an osteoclast-secreted protein with a positive impact on the osteogenesis of stromal cells, and deletion of *Cthrc1* in osteoclasts leads to osteoporosis.^24^ However, Jin et al. showed that CTHRC1 is not expressed in osteoclasts, but in osteoblasts and osteocytes, and *Cthrc1* null mice not only exhibit reduced bone formation but also show enhanced bone resorption with enhanced osteoclast number and activity.^28^ In our study, we found that CTHRC1 was also expressed by USCs; moreover, this protein was enriched in USC-EVs and required for the USC-EV-induced promotion of osteogenesis and inhibition of osteoclast formation. USC-EVs also contained highly abundant OPG protein, which is an evolutionarily conserved secretory glycoprotein that can inhibit osteoclastogenesis by neutralizing RANKL (a key mediator of osteoclast formation and function).^29,48^ Further studies showed that OPG partially mediated the anti-osteoclastic and bone-protective effects of USC-EVs. Our results suggest that USC-EVs deliver functional CTHRC1 and OPG to enhance osteogenesis and suppress osteoclastogenesis, thus enhancing bone mass and strength. However, the detailed processes and underlying mechanism regarding how CTHRC1 and OPG proteins are sorted into USC-EVs and then reused by recipient cells and tissues still require further investigation. Furthermore, when we explored the roles of CTHRC1 and OPG in USC-EV-induced pro-osteogenic and/or anti-osteoclastic effects, we did not perform experiments to comprehensively and systematically assess whether RNA interference (RNAi) by shOPG or shCTHRC1 in USCs could influence the loading of other molecules, except for OPG and CTHRC1, into USC-EVs. Although numerous studies have exploited RNAi to interfere with the expression of specific genes in cells and used the EVs from these cells to assess the role of the specific genes in EV-mediated regulatory effects,^49–53^ RNAi might have the potential to affect the sorting of other proteins or RNA into EVs through some unknown mechanisms and thereby alter the activity of EVs, which warrants further exploration.

Notably, the downregulation of either CTHRC1 or OPG did not entirely block the bone-protective effects of USC-EVs, which suggests that other factors may also be implicated in mediating these processes, in accordance with the fact that USC-EVs also contained other pro-osteogenic or anti-osteoclastic proteins. Thus, the regulatory effects of USC-EVs on recipient cells are mediated by the transfer of multiple signaling molecules, rather than just by a single factor or two factors. An adequate blood supply is important for bone formation and maintenance,^18,54^ and we previously confirmed that USC-EVs are able to transfer pro-angiogenic proteins to endothelial cells and thereby promote angiogenesis.^9^ It is possible that the stimulation of angiogenesis may also contribute to USC-EVs-mediated anti-osteoporotic effects, opening a new therapeutic mechanism for the USC-EVs-based treatment of osteoporosis.

## Materials and methods

### Identification of human USCs and USC-EVs

The approval for this work was obtained from the Ethics Committee at Xiangya Hospital of Central South University and written informed consent was obtained from each donor. Urine specimens (50 mL for each sample) were harvested from seven healthy donors (one girl and two boys of 3−5-years old, two men and one woman of 24−28 years old, and a 65-year-old healthy man) and two osteoporotic postmenopausal women aged 63 and 78 years, respectively. The inclusion criteria were as follows: no infectious diseases (such as urinary tract infection, genital infection, etc.), no chronic diseases (such as diabetes, cardiocerebral vascular diseases, hypertension, kidney disease, tumor, etc.) and no use of specific medications during the past month. The medical examination results of the renal and hepatic function tests as well as the urine routine tests for these donors are shown in Supplementary Table 1.

The isolation of USCs by a simple centrifugation procedure and the culture conditions of USCs were described in detail in our recently published study.^9^ Briefly, a urine specimen in a 50 -mL centrifuge tube was centrifuged at room temperature for 10 min at 400 × *g*. The supernatant was then aspirated and only 1 mL of liquid was left in the centrifuge tube. After gently resuspending, the pellet in the remaining 1 mL of urine, 10 mL PBS containing 1% antibiotic–antimycotic (100 × stock; Gibco, USA) was added to the tube and mixed well. After centrifugation for 10 min at 200 × *g*, the supernatant was then removed, leaving only ~0.2 mL of liquid plus the cell pellet. Three milliliters of primary medium, which contained DMEM/F-12 (Gibco), a REGM SingleQuot kit (Lonza, Walkersville, MD, USA), 1% antibiotic–antimycotic (Gibco), and 10% fetal bovine serum (FBS; Gibco), were added to the cell pellet and mixed gently. The cell suspension was added to three wells (1 mL/well) of a 12-well plate. After incubation at 37 °C for 48 h, 1 mL of primary medium was added to each well. At 96 h later, 1 mL of primary medium was discarded and replaced with 1 mL of fresh proliferation medium, which contained mixed DMEM/F-12 and REGM Bullet kit (Lonza) at a ratio of 1:1, 10% FBS (Gibco), 1% NEAA (Gibco), 1% GlutaMAX (Gibco), 1% antibiotic–antimycotic (Gibco), 5 ng·mL^−1^ PDGF-BB (Peprotech, USA), 5 ng·mL^−1^ bFGF (Peprotech), and 5 ng·mL^−1^ EGF (Peprotech). The whole medium was then renewed with proliferation medium every 2 days. The cells were passaged when reaching 80%–90% confluence. USCs at passages 2–6 were used for subsequent experiments.

The expression of CD29, CD34, CD44, CD45, CD73, and CD90 on USCs was tested by flow-cytometric assay. Negative control cells were incubated with the isotype control antibodies. The antibodies were purchased from BD Biosciences (USA). Multipotent differentiation potential of USCs toward osteogenesis, chondrogenesis, and adipogenesis was assessed by culturing USCs in osteogenic, chondrogenic, and adipogenic medium (Cyagen Biosciences, Guangzhou, China) according to the manufacturer’s protocol. ARS, Oil Red O, and Alcian Blue staining were conducted to test the formation of calcium nodules, lipid droplets, and extracellular matrix proteoglycans on day 14, 21, and 28, respectively.

USC-EVs were obtained from USC-conditioned medium with Exoquick-TC Exosome Precipitation Solution (System Biosciences, USA), as previously described.^9^ The USC-EV pellets were dissolved in PBS, and their protein concentration was assessed using a BCA Protein Assay Kit (Thermo Fisher Scientific, USA). The numbers of EVs in 100 μg of USC-EV samples (called E1, E2, E3, E4, and E5, respectively) prepared from five different batches were assessed using an EXOCET Exosome Quantitation kit (Systems Biosciences). The size and morphology of USC-EVs were observed under a transmission electron microscope. The expression of TSG101, CD9, CD63, and CD81 on USC-EVs was detected by western blotting. All procedures were the same as described in detail in our recently published study.^9^

### Animals and treatments

Approval for animal care and experiments was obtained from the Ethics Committee of Xiangya Hospital of Central South University. For EV treatment, 100 μg of USC-EVs (dissolved in 100 μL PBS) or an equal volume of the vehicle (PBS) was injected into the mouse via the tail vein.

To test the tissue distribution of USC-EVs, the vesicles were labeled with the lipophilic dye DIR iodide (Santa Cruz Biotechnology, Santa Cruz, USA) for ex vivo fluorescent imaging or labeled with DiO (Invitrogen, Carlsbad, USA) for fluorescence microscope observation. Twelve C57BL/6 female mice (8–10-week-old; weighing 20 g–25 g) were anesthetized and subjected to bilateral OVX, as described previously.^18^ One week later, OVX mice were divided into four groups. Six mice were injected with DiR-labeled USC-EVs or PBS. The other six mice were injected with an equal amount of DiO-labeled USC-EVs or vehicle. No mice died after the OVX surgery or EV treatments. The mice injected with DiR- or DiO-labeled USC-EVs or vehicle for 3 h were anesthetized and killed to collect the liver, heart, lungs, spleen, kidneys, brain, femurs, and tibias. The samples were then processed for ex vivo fluorescent imaging or fluorescence microscope observation. All samples were kept away from light during the experiment.

To test the effects of USC-EVs from 28-year-old healthy adult woman-derived USCs on postmenopausal osteoporosis, thirty 8-10-week-old C57BL/6 female mice underwent either OVX (*n* = 20) or a sham surgery (Sham; *n* = 10). One week later, 100 μL of vehicle (PBS; *n* = 10) or USC-EVs (*n* = 10) were injected into OVX mice. The mice in the Sham group (*n* = 10) were also injected with 100 μL of PBS. The treatments were conducted once a week. No mice died after the surgery or EV treatments. Two months later, all mice were anesthetized. Blood samples were obtained by enucleation of the eyeball, and the mice were then killed. Serum samples were obtained by centrifugation at 1 000 *g* for 15 min and stored at −80 °C until analyses. For ELISA tests, five serum samples were randomly selected and thawed for testing. All uteri were isolated and weighed. All spleen samples were obtained, and three spleen samples in each group were randomly selected for H&E staining. All right femora were collected for μCT analysis. Five left femora per group were randomly selected for the three-point bending test, and the other five left femora in each group were processed for immunohistochemical staining for OCN and TRAP staining. Three bone samples per group and three sequential sections per sample were initially randomly selected for photographing and quantification. However, the bone tissues from some mice were very easy to detach from the glass slides before or during the staining. Once irretrievable tissue detachment occurred, we selected bone samples that showed better morphological integrity on glass slides for photographing and quantification. No other obvious differences were observed between the selected bone samples and nonselected bone samples (the same as below).

To test the impact of USC-EVs on senile osteoporosis, sixteen 16-month-old C57BL/6 mice (*n* = 8 per group) were administered USC-EVs from 28-year-old healthy adult woman-derived USCs or PBS (control group) once a week. One mouse in the control group and two mice in the USC-EV group died of unknown causes after two times of PBS or EV injections. All live mice (*n* = 6–7 per group) were killed after treatment for 3 months, and their blood, spleen, and femora were obtained. Five serum samples per group were randomly selected for ELISA. All right femora were subjected to μCT analysis. Five left femora per group were randomly selected for the three-point bending test.

To test the effects of USC-EVs on disuse osteoporosis, 3-month-old C57BL/6 mice underwent hind-limb unloading by TS (*n* = 21) or hind-limb loading (control group; *n* = 10). One hundred microliters of PBS or USC-EVs (100 μg in 100 μL PBS) from 28-year-old healthy adult woman-derived USC were injected intravenously into the TS mice (*n* = 11 in PBS group and *n* = 10 in USC-EV group) twice a week for 3 weeks. Mice in the control group (*n* = 10) were also injected with 100 μL of PBS. One mouse in the control group died of unknown causes after one round of PBS injections. All live mice (*n* = 9–11 group) were killed after treatment for 3 weeks, and their blood, spleen, and femora were obtained. Five serum samples per group were randomly selected for ELISA. All right femora were obtained for μCT analysis. Five left femora per group were randomly selected for the three-point bending test.

To assess the effects of USC-EVs from three different donors, fifty 8- to10-week-old C57BL/6 female mice underwent OVX (*n* = 40) or a sham surgery (*n* = 10). Five mice died after OVX. The live OVX mice were injected with PBS (*n* = 10) or different donor-derived USC-EVs (Y-USC-EVs: *n* = 9; A-USC-EVs: *n* = 8; O-USC-EVs: *n* = 8). Sham mice were also treated with PBS. The treatments were conducted once a week. In the OVX + A-USC-EV group and the OVX + O-USC-EV group, a mouse from each group died of unknown causes after treatment for two weeks. All live mice (*n* = 7–10 per group) were killed after treatment for 2 months, and their blood, uteri, spleen, and femora were obtained. All uteri were isolated and weighed. All right femora were obtained for μCT analysis and then for a three-point bending test. All left femora were processed and embedded in paraffin. Three bone samples in each group were initially randomly selected, and three sequential sections per sample were cut for OCN and TRAP staining. Once irretrievable tissue detachment occurred, we continued to randomly select other bone samples from the remaining samples for staining and quantification.

To test the role of CTHRC1 and OPG in USC-EV-induced regulatory effects in osteoporotic mice, forty 8- to10-week-old C57BL/6 female mice were subjected to OVX (*n* = 32) or sham surgery (*n* = 8). Three mice died after OVX. One week later, the live OVX mice were injected with PBS (*n* = 8 in this group) or USC-EVs (*n* = 7 per group) from control USCs or CTHRC1- or OPG-knockdown USCs. Sham mice were injected with PBS. The treatments were conducted once a week. A mouse treated with USC-EVs from CTHRC1-knockdown USCs died of unknown causes. Two months later, all live mice (*n* = 6–8 per group) were killed, and their blood, uteri, spleen, and femora were obtained. All uteri were isolated and weighed. All right femora were harvested for μCT analysis and then for three-point bending test. All left femora were processed and embedded in paraffin. Three bone samples in each group were randomly selected based on the above-described principles and processed for OCN and TRAP staining.

To test the influence of USC-EVs on dynamic bone formation, nine 8- to 10-week-old C57BL/6 female mice underwent OVX (*n* = 6) or a sham surgery (*n* = 3). One week later, OVX mice (*n* = 3 per group) were treated with PBS or USC-EVs once a week for 2 months. Sham mice were injected with PBS. All mice received an intraperitoneal injection of 0.1% calcein in PBS at 10 and 3 days before euthanasia. Femora were collected and processed for detecting calcein double labeling under a fluorescence microscope. No mice died during the experiment.

To explore the role of CTHRC1 and OPG in USC-EV-induced modulation of dynamic bone formation, fifteen 8- to 10-week-old C57BL/6 female mice underwent OVX (*n* = 12) or a sham operation (*n* = 3). One week later, OVX mice (*n* = 3 per group) were injected with PBS or USC-EVs from CTHRC1- or OPG-knockdown USCs or control USCs once a week for 2 months. Sham mice were injected with PBS. All mice received an intraperitoneal injection of 0.1% calcein in PBS at 10 and 3 days before euthanasia. Femora were collected and processed for detecting calcein double labeling under a fluorescence microscope. No mice died in the experiment.

### Tissue distribution of USC-EVs

For ex vivo fluorescent imaging, the obtained fresh tissues from the mice treated with DiR-labeled USC-EVs or vehicle were scanned by a fluorescence tomography imaging system (FMT-4000; PerkinElmer, USA). For fluorescence microscopy observation, the tissues from mice treated with DiO-labeled USC-EVs or vehicle were fixed with 4% paraformaldehyde (PFA) for 24 h. Bone tissues were then decalcified in EDTA (0.5 mol·L^−1^) with shaking at 4 ℃ for 1 week. All samples were immersed in 30% sucrose aqueous solution for 2 days at room temperature for dehydration, exposed to liquid nitrogen for a few seconds, and then embedded in OCT compound (Tissue-Tek, Torrance, USA). Then, 20-μm-thick bone slices were made, and the other tissues were sectioned into 10-μm-thick slices. DAPI (0.5 µg·mL^−1^; Invitrogen) was applied to stain the nuclei. Images were obtained using a Zeiss ApoTome fluorescence microscope (Germany).

### μCT analysis

Femur samples were fixed with 4% PFA for 2 days, transferred into PBS, and then scanned by μCT (Skyscan 1176; Skycan, Aartselaar, Belgium). The voltage, current, and resolution were set to 50 kV, 400 μA, and 8.88 μm per pixel, respectively. For the trabecular bone, the selected areas for analysis were the regions between 0.45 mm and 0.90 mm proximal to the growth plate in the distal femurs. Tb. BV/TV, Tb. Th, Tb. N, and Tb. Sp were measured. For cortical bone, the region of interest was 5% of the femoral length in mid-diaphysis of the femur to evaluate Ps. Pm, Es. Pm, and Ct. Th. The μCT analysis was conducted by a technician blinded to the study design and then analyzed by a main author in this paper blinded to the nature of the samples until data gathering was completed.

### Measurement of femur biomechanical parameters

Three-point bending test was conducted to assess the femur strength using a computer-controlled mechanical testing machine (WD-D1; Shanghai Zhuoji Instruments Co. Ltd., China). The loading point is in the middle of the femur, with two fulcrums spaced 8 mm and a loading speed of 5 mm·min^−1^. Biomechanical data were obtained from the load-deformation curves, and the ultimate load value of the femur (N) was calculated.

### Histological, immunohistochemical, and histomorphometric analyses

Femur samples were fixed with 4% PFA for 2 days, decalcified in 18% EDTA (pH = 7.4) for 1 week, dehydrated using increasing concentrations of ethanol and embedded in paraffin. Subsequently, 5 μm-thick bone sections were made and stained with OCN antibody (Abcam, Cambridge, Britain) or TRAP reagent (Sigma, St. Louis, MO, USA) as described previously.^18,32^ The sections were examined using a microscope (CX31; Olympus, Tokyo, Japan). Positively stained osteoblasts and osteoclasts were quantified and normalized to the number per millimeter of adjacent bone surface (N·mm^−1^).

To test dynamic bone formation, mice in different groups received intraperitoneal injection of 0.1% calcein (10 mg·kg^−1^ body weight; Sigma) in PBS at 10 and 3 days before euthanasia. Femora were obtained after killing the mice. The samples were then fixed with 4% PFA for 48 h, dehydrated in ethanol, and embedded in methyl methacrylate. The 60-μm-thick bone slices were obtained using a microtome. Calcein double labeling was examined under a fluorescence microscope (Leica). The MAR of trabecular bone was measured using Image-Pro Plus 6 software.

### ELISA

The serum concentrations of OCN, CTX-I, TNF-α, IL-6, IL-1α, and IL-1β were tested by using commercial ELISA kits from Elabscience (Wuhan, China) or MultiSciences Biotech Co., Ltd. (Hangzhou, China).

### Culture of MSCs and osteoclast progenitors

MSCs from C57BL/6-Tg(UBC-GFP)30Scha/J mice (Jackson Laboratory) and RAW264.7 osteoclast progenitors (ATCC, Rockville, USA) were cultured in high glucose DMEM (Gibco) containing streptomycin (100 U·mL^−1^; Gibco), penicillin (100 U·mL^−1^; Gibco) and 10% FBS (Gibco). The cells were maintained at 37 °C with 5% CO_2_ and passaged after becoming 80% confluent.

### EV uptake assay

USCs were stained red with Vybrant DiL cell-labeling solutions (Thermo Fisher Scientific), washed twice with PBS, and resuspended in EV-free complete medium. The DiL-labeled cells were then placed in culture flasks and cultured for 2 days. These cell-derived USC-EVs were isolated and incubated with MSCs or RAW264.7 cells at 37 °C. Three hours later, MSCs and RAW264.7 cells were fixed with 4% PFA for 15 min after washing, stained with DAPI (0.5 µg·mL^−1^; Invitrogen), and then observed under a fluorescence microscope.

To explore OPG location after the internalization of USC-EVs into recipient cells, USCs were transfected with plasmids expressing OPG-EGFP fusion protein or with control plasmids (Shanghai GeneChem Co. Ltd., China) using Lipofectamine 3000 (Invitrogen). EVs from USCs expressing OPG-EGFP (USC-EVs^OPG-EGFP^) or EVs from USCs transfected with control plasmids (USC-EVs^Con^) were harvested. USC-EVs^OPG-EGFP^ or USC-EVs^Con^ were added to the medium of RAW264.7 cells. Three hours later, the treated cells were fixed with 4% PFA for 15 min after washing, stained with DAPI and examined under a fluorescence microscope.

### Osteogenic differentiation assay

After culture in 48-well plates until 80% confluence, the MSCs were washed with PBS and then incubated with osteogenic differentiation medium containing 20, 100, or 300 μg·mL^−1^ USC-EVs or vehicle (PBS). Half of the induction medium was replaced by an equivalent volume of fresh osteogenic medium supplemented with USC-EVs at a final dose of 20, 100, or 300 μg·mL^−1^ or with vehicle (PBS) every other day. The negative control cells were incubated in DMEM + 10% FBS + vehicle. After 7 days of osteogenic differentiation (USC-EVs were added at day 1, 3, and 5), the total RNA from cultured MSCs was extracted, and the expression of *Alp*, *Ocn*, and *Runx2* was assayed by qRT-PCR. Fourteen days after osteogenic induction (USC-EVs were added at day 1, 3, 5, 7, 9, 11, and 13), the cultures were stained with ARS solution (Solarbio, Beijing, China) to evaluate the cell matrix mineralization.

### Osteoclastic differentiation assay

The osteoclast progenitor RAW264.7 cells were plated in 48-well culture plates (1.0 × 10^4^/well) and incubated overnight. The medium was changed to fresh complete DMEM containing 100 ng·mL^−1^ RANKL and 20, 100, or 300 μg·mL^−1^ USC-EVs or vehicle (PBS). The half medium was replaced by RANKL-containing fresh medium supplemented with USC-EVs at a final dose of 20, 100, or 300 μg·mL^−1^ or with vehicle (PBS) every other day. The negative control cells were grown in complete DMEM + vehicle. After 8 days of induction (USC-EVs were added at day 1, 3, 5, and 7), the cells were collected for assessing the mRNA levels of *Trap*, *Mmp9*, *Ctsk*, *Oscar*, *Ocstamp*, and *Atp6v0d2* by qRT-PCR analysis or stained for TRAP (Sigma). The numbers of TRAP^+^ osteoclasts (> 3 nuclei) in each well were counted under a microscope (Leica).

### Proteomic analysis

The above-mentioned healthy adult woman-derived USCs (three biological replicates called C1, C2, and C3) and their secreted USC-EVs (E1, E2, and E3) were collected and processed for proteomic analysis (Jingjie PTM BioLab, Hangzhou, China). The procedures had been described in detail in our recently published study.^9^

### Inhibition of CTHRC1 and OPG

Con shRNA and lentiviral shRNAs targeting human CTHRC1 (shCTHRC1 #1, shCTHRC1 #4, and shCTHRC1 #5) and OPG (shOPG #2, shOPG #4, and shOPG #5) were purchased from Cyagen Biosciences (Guangzhou, China). The packaging of viruses was also conducted by Cyagen Biosciences. For transfection, USCs were incubated in retroviral supernatant containing polybrene (10 μg·mL^−1^; Cyagen) for 24 h. Then, the medium was changed by fresh complete medium. At 72 h later, the cells were selected by puromycin (2.5 μg·mL^−1^; Sigma). The shRNA sequences were as follows: shCTHRC1 #1: 5′-TCTTCCCATTGAAGCTATAATCTCGAGATTATAGCTTCAATGGGAAGA-3′ shCTHRC1 #4: 5′-GCGTTGGTATTTCACATTCAACTCGA GTTGAATGTGAAATACCAACGC-3′ shCTHRC1 #5: 5′-CGCATCATTATTGAAGAACTACTCGAGTAGTTCTTCAATAATGATGCG-3′ shOPG #2: 5′-GCTCAGTTTGTGGCGAATAAACTCGAGTTTATTCGCCACAAACTGAGC-3′ shOPG #4: 5′-TAACCAGGTCCAATCAG TAAACTCGAGTTTACTGATTGGACCTGGTTA-3′ shOPG #5: 5′-ATGCAACACACGACAACATATCTCGAGATATGTTGTCGTGTGTTGCAT-3′ Con shRNA: 5′-CCTAAGGTTAAGTCGCCCTCGCTCGAGCGAGGGCGACTTAACCTTAGG-3′.

### qRT-PCR

After isolation of the total cellular RNA by TRIzol Reagent (Invitrogen), 1 μg of the total RNA was used for reverse transcription using a commercial kit (Fermentas, Burlington, Canada). qRT-PCR reactions (20 μL) were conducted on an ABI PRISM^®^ 7900HT System (Applied Biosystems, USA). Relative mRNA levels were calculated by the comparative Ct (2^–ΔΔCT^) method using GAPDH or β-actin for normalization. Primer sequences were as follows: *mouse-Ocn*: forward, 5′-CTGACCTCACAGATCCCAAGC-3′ and reverse, 5′-TGGTCTGATAGCTCGTCACAAG-3′ *mouse-Alp*: forward, 5′-CCAACTCTTTTGTGCCAGAGA−3′ and reverse, 5′-GGCTACATTGGTGTTGAGCTTTT-3′ *mouse-Runx2*: forward, 5′-GACTGTGGTTACCGTCATGGC-3′ and reverse, 5′-ACTTGGTTTTTCATAACAGCGGA-3′ *mouse-Trap*: forward, 5′-TGGTCCAGGAGCTTAACTGC-3′ and reverse, 5′-GTCAGGAGTGGGAGCCATATG-3′ *mouse-Mmp9*: forward, 5′-ACCCGAAGCGGACATT-3′ and reverse, 5′-GGCATCTCCCTGAACG-3′ *mouse-Ctsk*: forward, 5′-g-3′ and reverse, 5′-CTGGAAGCACCAACGA-3′ *mouse-Oscar*: forward, 5′-GGTCCTCATCTGCTTG-3’ and reverse, 5′-TATCTGGTGGAGTCT GG-3′ *mouse-Ocstamp*: forward, 5′-GGGCTACTGGCATTGCTCTTAGT-3′ and reverse, 5′-CCAGAACCTTATATGAGGCGTCA-3′ *mouse-Atp6v0d2*: forward, 5′-AGCAAAGAAGACAGGGAG-3′ and reverse, 5′-CAGCGTCAAACAAAGG-3′ *mouse-β-actin*: forward, 5′-GGCTGTATTCCCCTCCATCG-3′ and reverse, 5′-CCAGTTGGTAACAATGCCATGT-3′ *human-Cthrc1*: 5′-CAATGGCATTCCGGGTACAC-3′ and reverse, 5′-GTACACTCCGCAATTTTCCCAA-3′ *human-Opg*: 5′-GCGCTCGTGTTTCTGGACA-3′ and reverse, 5′-AGTATAGACACTCGTCACTGGTG-3′ *human-GAPDH*: forward, 5′-ATCCCATCACCATCTTCC-3′ and reverse, 5′-GAGTCCTTCCACGATACCA-3′.

### Western blot analysis

After separation by SDS-PAGE, cells or EVs protein extracts (30 μg) were blotted on PVDF membranes. The membranes were blocked in 5% milk for 60 min and incubated overnight with specific antibodies to TSG101 (1:1 000; ProteinTech, Chicago, USA), CD81 (1:500; Santa Cruz), CD63 (1:500; Santa Cruz), CD9 (1:500; Santa Cruz), CTHRC1 (1:500; Absin, Shanghai, China), OPG (1:1 000; R&D Systems, Minneapolis, USA) or SFRP1 (1:1 000; R&D Systems). Then, the membranes were incubated for 1 h at room temperature with the respective secondary antibodies (1:5 000; Cell Signaling Technology, Danvers, USA). The blots were then detected with an enhanced chemiluminescence kit (Thermo Fisher Scientific).

### Statistical analysis

The data are shown as the mean ± SD and analyzed using GraphPad Prism software. The unpaired, two-tailed Student’s *t* test was conducted to compare differences between two groups. Multiple-group comparisons were assessed by one-way analysis of variance (ANOVA) with Bonferroni’s post hoc test. *P* < 0.05 was considered statistically significant.

## Supplementary information

Supplementary information

Supplementary Table 1
