# Supplementary material for: Extracellular vesicles from human urine-derived stem cells prevent osteoporosis by transferring CTHRC1 and OPG
Source: Bone Res. 2019 Jun 26;7:18. doi: 10.1038/s41413-019-0056-9 (PMC6594995; doi:10.1038/s41413-019-0056-9)
Supplement: Supplementary file 1 — Supplementary information [file 41413_2019_56_MOESM1_ESM.docx]

**Supplementary Materials for**

**Extracellular vesicles from human urine-derived stem cells prevent osteoporosis by transferring CTHRC1 and OPG**

Chun-Yuan Chen†, Shan-Shan Rao†, Yi-Juan Tan, Ming-Jie Luo, Xiong-Ke Hu, Hao Yin, Jie Huang, Yin Hu, Zhong-Wei Luo, Zheng-Zhao Liu, Zhen-Xing Wang, Jia Cao, Yi-Wei Liu, Hong-Ming Li, Yang Chen, Wei Du, Jiang-Hua Liu, Yan Zhang, Tuan-Hui Chen, Hao-Ming Liu, Ben Wu, Tao Yue, Yi-Yi Wang, Kun Xia, Peng-Fei Lei, Si-Yuan Tang, Hui Xie**^*^**

†Chun-Yuan Chen and Shan-Shan Rao contributed equally to this work.

*Correspondence to: huixie@csu.edu.cn

**
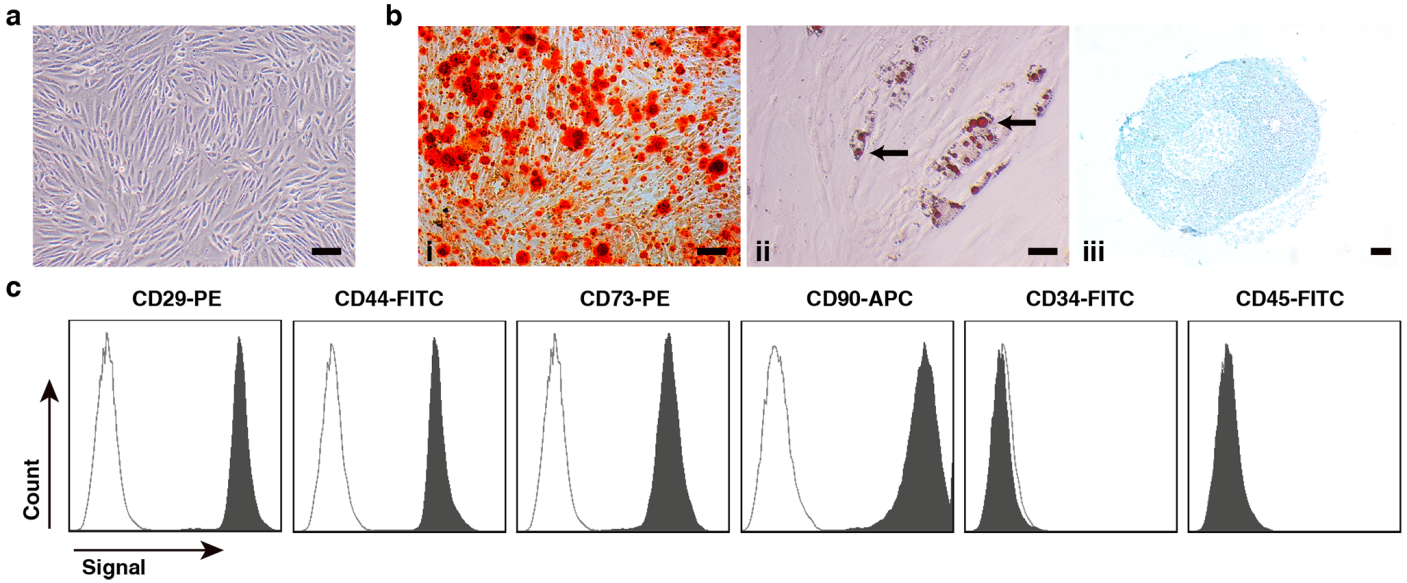
**

**Supplementary Figure 1.** **Characterization of USCs. (a)** Morphology of USCs under light microscope. Scale bar: 100 μm. **(b)** USCs were able to differentiate into osteoblasts, adipocytes and chondrocytes, as evidenced by ARS staining (**i**; mineralization areas showing red staining; Scale bar: 100 μm), Oil Red O staining (**ii**; Scale bar: 50 μm) and Alcian Blue staining (**iii**; chondrocytes showing blue staining; Scale bar: 200 μm), respectively. Black arrows indicate red-stained lipid droplets. **(c)** Cell surface markers on USCs were assessed by flow cytometry analysis. The test samples are illustrated as solid gray curves and the isotype controls are illustrated as blank curves.

**
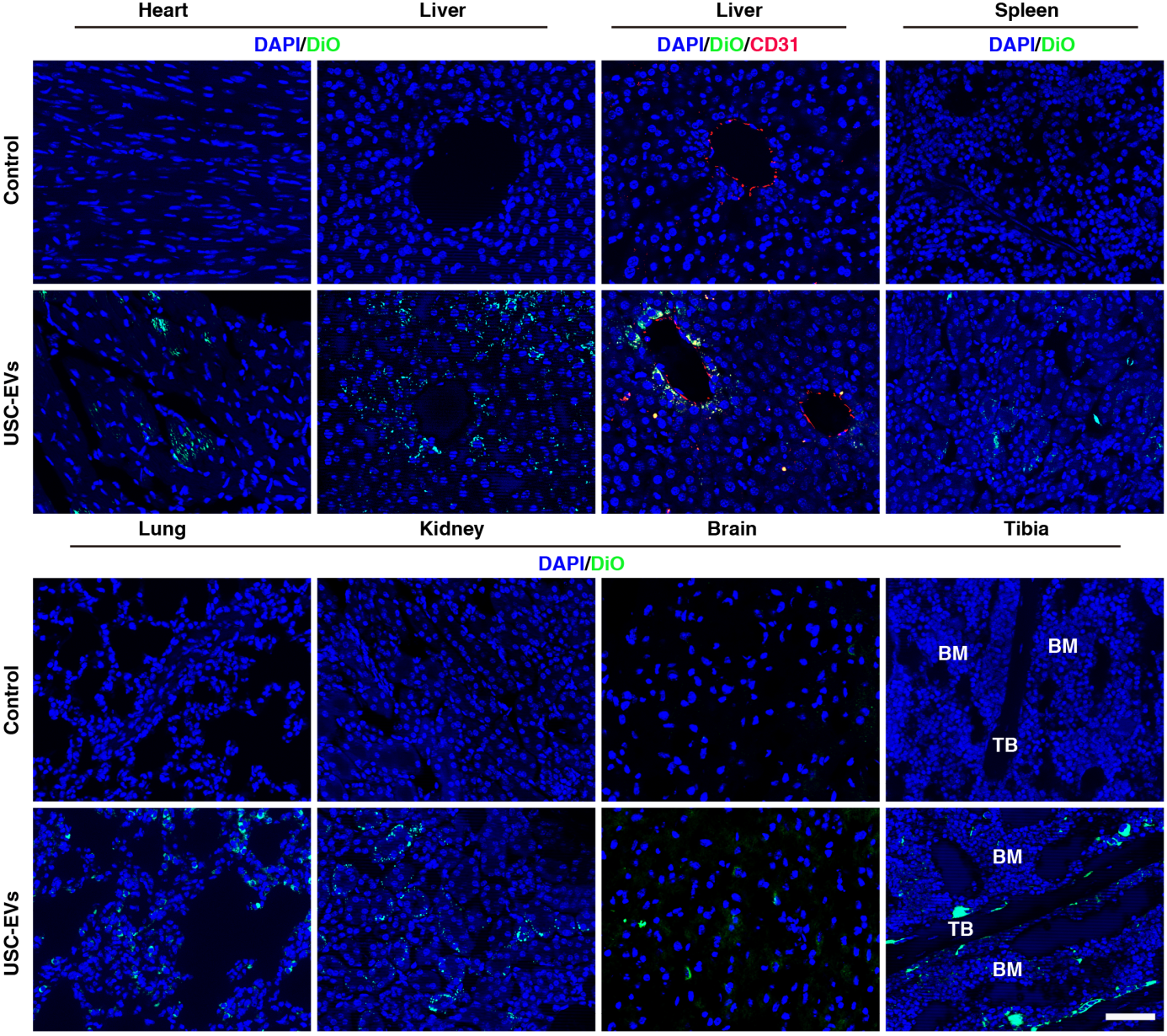
**

**Supplementary Figure 2.** **Distribution of DiO-labeled USC-EVs in** **heart, liver, spleen, lung, kidney, brain and tibia of OVX mice.** Representative images of heart, liver, spleen, lung, kidney, brain and tibia sections from mice intravenously injected with vehicle (Control) or DiO-labeled USC-EVs for 3 h. For the liver tissues, the section was co-stained with an endothelial marker CD31 (red signals), in order to detect endothelial cells. TB: trabecular bone; BM: bone marrow. Scale bar: 50 μm.


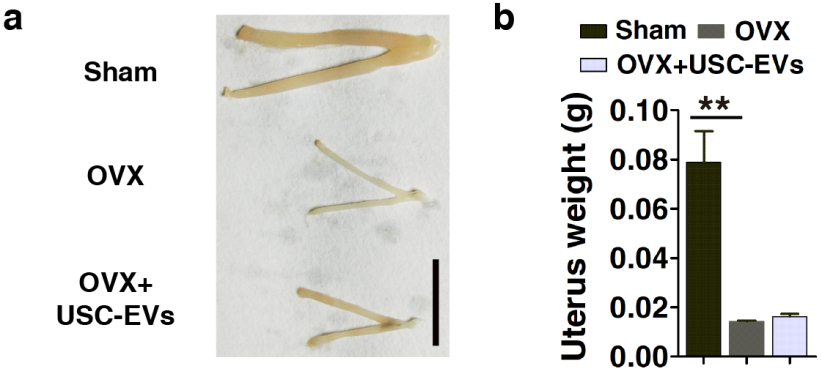


**Supplementary Figure 3. OVX mice exhibit decreased uterus size and** **uterus weight**. **(a-b)** Representative images **(a)** and quantitative analysis of weight **(b)** of uteruses from Sham, OVX and OVX + USC-EVs mice. Scale bar: 1 cm. *n* = 10 *per* group. Data are shown as mean ± SD. ******P* < 0.05, *******P* < 0.01, ********P* < 0.001.


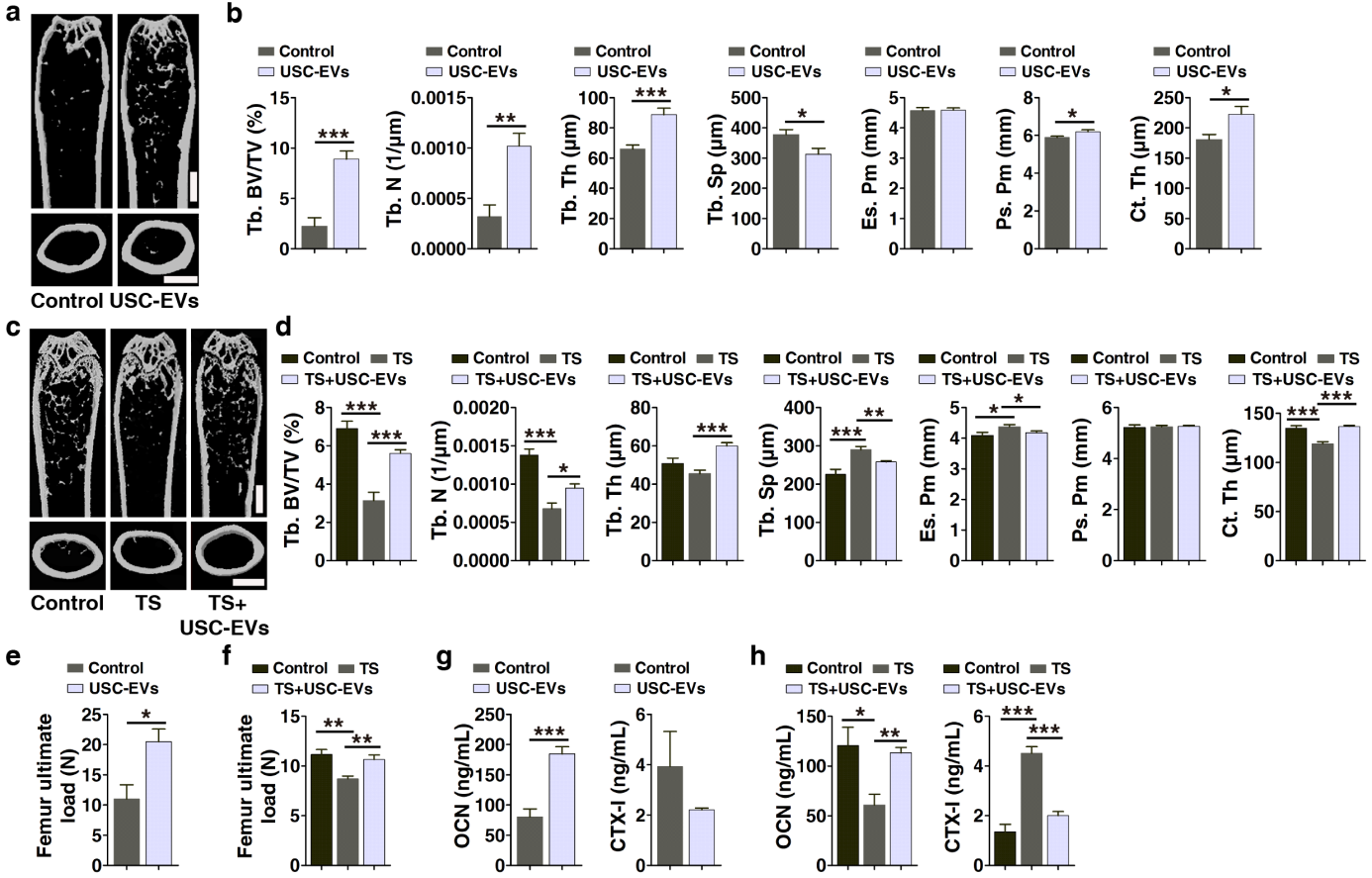


**Supplementary Figure 4. USC-EVs reverse the osteoporotic phenotypes in mouse models of senile osteoporosis and disuse osteoporosis. (a-b)** Representative μCT images (**a**) and quantitative μCT analysis of Tb. BV/TV, Tb. N, Tb. Th, Tb. Sp, Es. Pm, Ps. Pm and Ct. Th (**b**) of femora from vehicle (Control)- and USC-EVs-treated aging mice. Scale bars: 1 mm. *n* = 6-7 *per* group. (**c-d**) Representative μCT images **(c)** and quantitative μCT analysis of trabecular and cortical bone microarchitecture **(d)** in femora from Control, TS and TS + USC-EVs mice. TS: tail suspension. Scale bars: 1 mm. *n* = 9-11 *per* group. **(e-f)** Three-point bending measurement of ultimate load of the femur from mouse models of senile osteoporosis **(e)** and TS-induced hindlimb disuse osteoporosis **(f)**. *n* = 5 *per* group. **(g-h)** ELISA of the concentration of OCN and CTX-I in serum from mouse models of senile osteoporosis **(g)** and TS-induced hindlimb disuse osteoporosis **(h)**. *n* = 5 *per* group. Data are shown as mean ± SD. ******P* < 0.05, *******P* < 0.01, ********P* < 0.001.

**
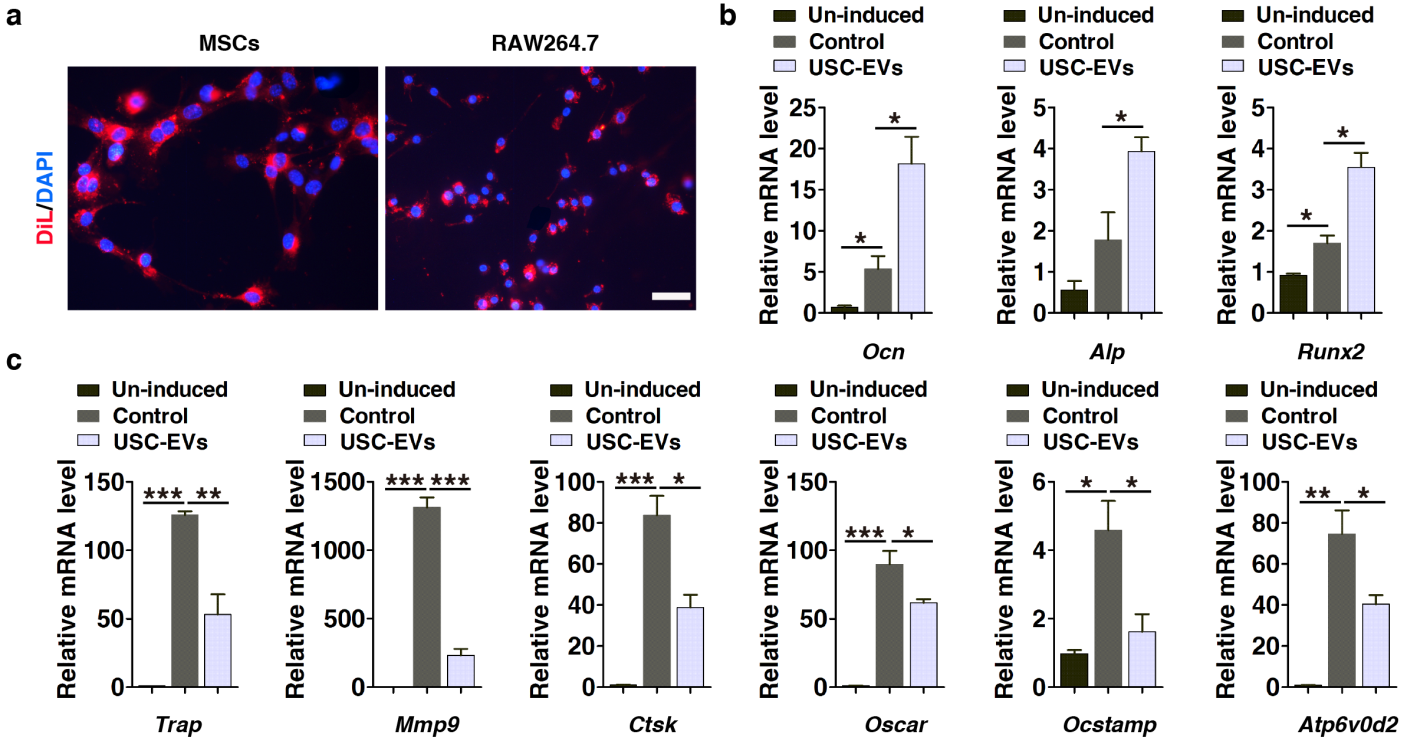
**

**Supplementary Figure 5.** **Internalization of USC-EVs into MSCs and RAW264.7 cells and their pro-osteogenic and anti-osteoclastic effects on recipient cells. (a)** Fluorescence microscopy analysis of DiL-labeled USC-EVs internalization by MSCs and RAW264.7 cells. The red-labeled USC-EVs were visible in the perinuclear region of recipient cells. Scale bar: 50 μm. **(b**-**c)** qRT-PCR analysis of the expression of genes related to osteogenesis **(b)** and osteoclastogenesis **(c)** in MSCs and RAW264.7 cells under osteogenic and osteoclastic differentiation, respectively. Data are shown as mean ± SD. ******P* < 0.05, *******P* < 0.01, ********P* < 0.001.

**
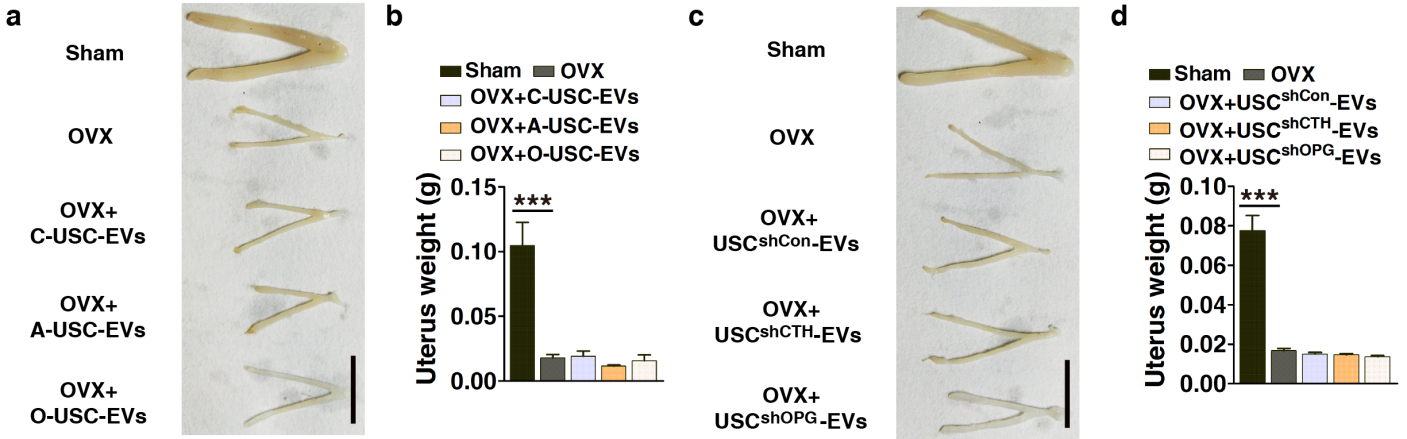
**

**Supplementary Figure 6. OVX mice exhibit decreased uterus size and uterus weight**. **(a-b)** Representative images **(a)** and quantitative analysis of weight **(b)** of uteruses from Sham, OVX, OVX + C-USC-EVs, OVX + A-USC-EVs and OVX + O-USC-EVs mice. Scale bar: 1 cm. *n* = 7-10 *per* group. **(c-d)** Representative images **(c)** and quantitative analysis of weight **(d)** of uteruses from Sham, OVX, OVX + USC^shCon^-EVs, OVX + USC^shCTHRC1 #4^-EVs and OVX + USC^shOPG #4^-EVs mice. Scale bar: 1 cm. *n* = 6-8 *per* group. Data are shown as mean ± SD. ******P* < 0.05, *******P* < 0.01, ********P* < 0.001.


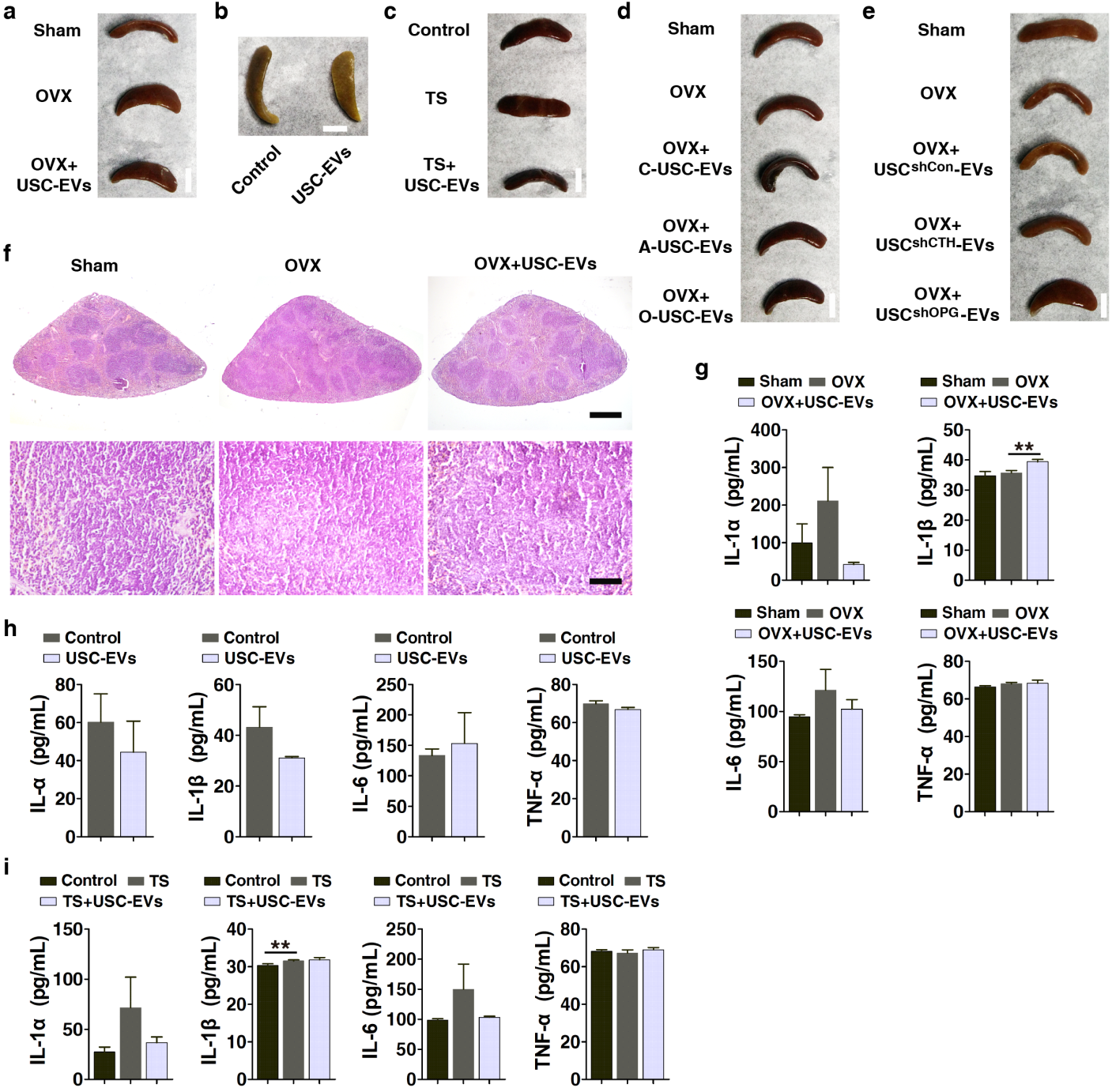


**Supplementary Figure 7. No obvious immune** **and inflammatory responses are induced in mice after USC-EVs injection.** **(a-e)** Gross view of spleen samples in postmenopausal osteoporosis (**a, d** and **e**), senile osteoporosis (**b**) and TS-induced hindlimb disuse osteoporosis (**c**) mouse models receiving different treatments. Scale bar: 0.5 cm. **(f)** Representative images of H&E-stained spleen sections from vehicle-treated Sham mice and vehicle or USC-EVs-treated OVX mice. Scale bar: 500 μm (above) and 50 μm (below). **(g-i)** ELISA of the serum concentration of IL-1α, IL-1β, IL-6 and TNF-α in postmenopausal osteoporosis (**g**), senile osteoporosis (**h**) and TS-induced hindlimb disuse osteoporosis (**i**) mouse models receiving different treatments. *n* = 5 *per* group. Data are shown as mean ± SD. ******P* < 0.05, *******P* < 0.01, ********P* < 0.001.


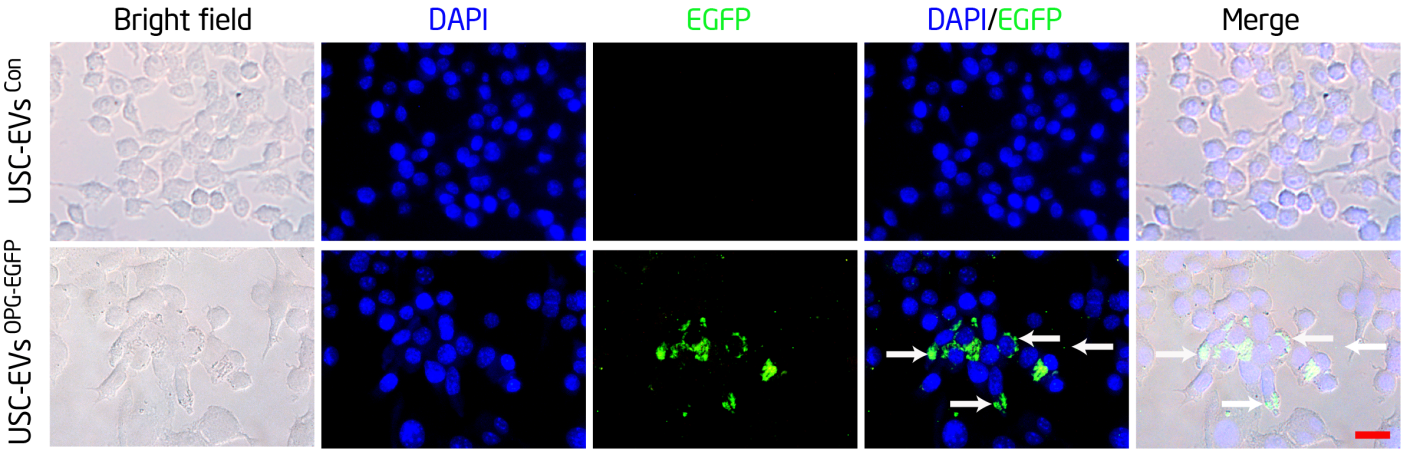


**Supplementary Figure 8. Location of OPG when USC-EVs are internalized by RAW264.7 cells.** The location of OPG-EGFP fusion protein was assessed by a fluorescence microscopy when RAW264.7 cells were incubated with USC-EVs^OPG-EGFP^ or USC-EVs^Con^ for 3 h. The EGFP signals were visible in the perinuclear region, cytoplasm, cell membrane as well as extracellular region of recipient cells. White arrows indicate OPG-EGFP. Scale bar: 25 μm.
